# Deciphering Cancer Therapy-Induced Cardiotoxicity in the Era of Spatial and Multi-Omics from Systemic Mechanisms to *In Situ* Microenvironments

**DOI:** 10.7150/jca.132588

**Published:** 2026-04-08

**Authors:** Cheng Gou, Qian Li, Ting Xiong, Shengzheng Lin, Kaining Wang, Jianhao Liu, Chuansong Xue

**Affiliations:** 1 Division of Cardiology, Guang'an People's Hospital, Guang'an, China.; 2 Department of Oncology, Shanghai Children's Medical Center, Shanghai Jiao Tong University School of Medicine, Shanghai, China.; 3 Internal Medicine of Three, Sanya Hospital of Traditional Chinese Medicine (Hainan Hospital, Guangzhou University of Chinese Medicine), Sanya, Hainan 572000, China.

**Keywords:** Cardio-oncology, Spatial Transcriptomics, Multi-omics, Artificial Intelligence, Precision Medicine

## Abstract

Cardio-oncology has emerged as a critical discipline addressing the cardiovascular sequelae of antineoplastic therapies. While traditional chemotherapy, targeted therapies, and immune checkpoint inhibitors (ICIs) have revolutionized cancer survival, they are frequently accompanied by cardiotoxicity ranging from asymptomatic left ventricular dysfunction to fulminant myocarditis. Current clinical strategies, relying heavily on echocardiography and serum biomarkers, often fail to detect early molecular perturbations or elucidate the complex multicellular interactions within the cardiac microenvironment. The advent of multi-omics technologies, genomics, transcriptomics, proteomics, and metabolomics, has provided systemic insights into these pathomechanisms. More recently, the integration of single-cell RNA sequencing (scRNA-seq) and spatial omics has enabled “*in situ*” visualization of cellular heterogeneity and intercellular communication, resolving the “blind spots” of bulk sequencing. This review synthesizes the current landscape of therapy-induced cardiotoxicity, highlights the limitations of conventional methodologies, and comprehensively examines how multi-dimensional omics strategies are reshaping our understanding of mechanisms, biomarker discovery, and precision cardioprotection.

## 1. Introduction

The therapeutic landscape of oncology has undergone a significant transformation in recent decades. With the introduction of potent chemotherapeutic agents, molecularly targeted therapies, and immune checkpoint inhibitors (ICIs), cancer is increasingly managed as a chronic condition rather than a terminal illness. However, this clinical success has given rise to a “survivor's paradox”: as cancer-specific mortality decreases, the incidence of competing risks, particularly from cardiovascular disease (CVD), has risen sharply 1. As a result, cardio-oncology has emerged to bridge the gap between oncological efficacy and cardiovascular safety.

Current management strategies, as guided by organizations such as the ESC, remain predominantly reactive instead of predictive 2. Diagnosis typically depends on functional imaging metrics like Left Ventricular Ejection Fraction (LVEF) or circulating biomarkers such as troponins. Although these markers are valuable, they tend to reflect established tissue damage, rather than capturing early, reversible molecular changes. Moreover, the heart is not a uniform organ; it is a complex, multicellular ecosystem consisting of cardiomyocytes, endothelial cells, fibroblasts, and immune cells. Traditional reductionist approaches, which often analyze the heart as a bulk tissue, fail to account for the intricate intercellular interactions and spatial heterogeneity that drive cardiotoxicity.

To overcome these limitations, a systems biology approach is essential. Multi-omics technologies, which integrate genomics, transcriptomics, proteomics, and metabolomics, provide a comprehensive understanding of the biological networks altered by anti-cancer therapies 3. Critically, these multidimensional data allow for the exploration of cardiotoxicity as a systemic syndrome rather than a localized cardiac event. Recent evidence highlights the role of inter-organ crosstalk, such as the heart-gut-microbiome axis 4 and the mobilization of pro-inflammatory progenitors from the bone marrow, in modulating myocardial resilience and driving systemic inflammatory states 5. Furthermore, the recent advancements in single-cell and spatial omics enable researchers to map these perturbations with unparalleled resolution, addressing not only “what” changes occur, but also “where” and “in which cell type” these changes take place. This review outlines the current understanding of cardiotoxicity mechanisms and illustrates how multi-dimensional omics strategies are paving the way for the next generation of precision cardio-oncology.

## 2. Spectrum of Cardiotoxicity: Evolving Mechanisms and Analytical Gaps

Understanding the transformative potential of omics requires a nuanced appreciation of the diverse pathological mechanisms underlying cardiotoxicity. The molecular drivers of cardiac damage vary significantly across therapeutic classes, each presenting unique challenges that traditional methodologies struggle to resolve.

### 2.1 The Anthracycline Paradigm: Beyond Oxidative Stress

Anthracyclines, such as doxorubicin, remain a cornerstone treatment for breast cancer, lymphomas, and sarcomas. However, their use is limited by cumulative, dose-dependent, and often irreversible cardiac damage. Doxorubicin-induced cardiomyopathy is a complex condition that can present with a wide range of symptoms, from acute to chronic forms. Acute cardiotoxicity occurs within 2-3 days of doxorubicin administration, often leading to myopericarditis, tachycardia, ECG alterations, and in some cases, left ventricular (LV) failure 6. Chronic cardiotoxicity, which develops over a more extended period (from 30 days to more than 10 years), can result in long-term heart failure. Approximately 1.7% of patients treated with doxorubicin develop chronic doxorubicin-induced cardiomyopathy (DCM), with incidence rates increasing significantly with higher doses (up to 36% for doses over 600 mg/m²) 7. The significant variability in the incidence of chronic DCM is influenced by factors such as patient age, underlying cardiovascular disease, and the total cumulative dose of the drug. Traditionally, this toxicity was attributed almost exclusively to the “ROS hypothesis,” in which iron-mediated reactive oxygen species induce lipid peroxidation and mitochondrial damage. However, the discovery that doxorubicin forms ternary complexes with DNA and Topoisomerase beta (Top2b), inducing DNA double-strand breaks and subsequent mitochondrial dysfunction, has fundamentally refined our understanding of the initiation phase 8.

Despite these mechanistic advances, a significant clinical conundrum persists: why do phenotypically similar patients exhibit vastly different tolerances to identical cumulative doses? This inter-individual variability suggests a complex, polygenic architecture of susceptibility that cannot be explained by single-gene polymorphisms alone. Furthermore, the downstream metabolic consequences of Top2b inhibition involve intricate shifts in mitochondrial substrate utilization and bioenergetics. Traditional biochemical assays, which target specific pathways in isolation, lack the breadth to capture these systemic metabolic reprogramming, highlighting the necessity for untargeted genomics and metabolomics to decode the full spectrum of susceptibility and damage. In terms of genetic factors, numerous studies have highlighted genetic variations that contribute to the risk of developing DCM. Variants in genes involved in oxidative stress, mitochondrial dysfunction, and apoptosis (e.g., *CYBA*) have been linked to an increased susceptibility to anthracycline-induced cardiotoxicity 9. In addition, rare protein-altering variants in established cardiomyopathy genes encoding sarcomeric/contractile components (e.g., *MYH7* and *TNNT2*) have been identified in chemotherapy-related cardiomyopathy cohorts and may contribute to individual susceptibility, potentially by lowering baseline myocardial functional reserve 10. Recent efforts have focused on identifying genetic biomarkers that could predict individual risk, paving the way for more personalized approaches to treatment. For example, genetic screening could identify patients who are more likely to develop cardiotoxicity, allowing clinicians to tailor chemotherapy regimens accordingly or to initiate cardioprotective strategies early.

### 2.2 Targeted Therapies: The Complexity of Signaling Interference

In contrast to the direct cytotoxicity of anthracyclines, targeted agents like HER2 inhibitors (e.g., trastuzumab) and Tyrosine Kinase Inhibitors (TKIs) typically induce a “Type II” toxicity characterized by cellular dysfunction rather than overt necrosis. Trastuzumab, for example, interrupts the neuregulin-1 (NRG1)/ErbB2 signaling axis, a critical pathway for cardiomyocyte survival and physiological stress adaptation 11. Bruton's Tyrosine Kinase (BTK) inhibitors, such as ibrutinib, are commonly associated with atrial fibrillation (AF). The mechanism of AF induction is related to the inhibition of PI3K-Akt signaling, which is involved in the heart's normal response to stress. The risk of AF is significantly lower with acalabrutinib, a second-generation BTK inhibitor, which suggests the involvement of additional signaling pathways 12. While this mechanism is well-defined, the clinical reality is often more complex due to the promiscuous nature of kinase inhibitors.

Kinase signaling networks are defined by redundancy and extensive crosstalk. Inhibiting a primary target often triggers compensatory activation of alternative pathways or unintended “off-target” effects on non-cardiac kinases. Standard protein analysis methods, such as Western blotting, are insufficient to map these dynamic, network-level phosphorylation changes. To fully understand how a TKI reshapes the cardiac signaling landscape, high-throughput phosphoproteomics is required to simultaneously monitor the activation states of hundreds of kinases, distinguishing adaptive responses from maladaptive toxicity.

### 2.3 Immunotherapy: The New Frontier of Inflammatory Toxicity

The rapid integration of immune checkpoint inhibitors (ICIs) targeting PD-1/PD-L1 or CTLA-4 has introduced immune-related myocarditis (ICI-M). ICI-induced myocarditis is rare, affecting approximately 1% of patients, but early pharmacovigilance and registry-based reports suggested a high case-fatality rate of 27-50% 13. However, these early estimates may be influenced by ascertainment and reporting bias toward severe or fatal cases. More recent prospective and contemporary cohort studies have reported variable mortality rates that depend on clinical severity, timeliness of recognition, and the immunosuppressive treatment protocol used. The prognosis of ICI-associated myocarditis is much worse compared to other types of myocarditis, with a significantly higher short-term mortality rate. Studies have also shown that cancer patients treated with ICIs have an 11-fold increased likelihood of developing myocarditis compared to those not treated with ICIs 14, 15. This severe prognosis is attributed to fatal cardiovascular complications such as arrhythmias and heart failure, or the interruption of effective cancer treatments. By blocking PD-1/PD-L1 or CTLA-4 checkpoints, these agents unleash T-cells to attack tumors but can inadvertently break peripheral tolerance, leading to autoreactive T-cell infiltration into the myocardium 16. Macrophages also play a central role in amplifying myocardial injury, releasing pro-inflammatory chemokines (e.g., CXCL9, CXCL10) that recruit additional effector cells and contributing to antigen presentation, which sustains the inflammatory cascade. Dysregulated dendritic cells (DCs), neutrophils, NK cells, and B cells further disrupt immune homeostasis through aberrant activation, impaired regulation, and crosstalk that perpetuates the pro-inflammatory microenvironment. Non-immune resident cells, particularly cardiac fibroblasts, actively reinforce this milieu by secreting cytokines and chemokines, undergoing inflammatory activation, and engaging in bidirectional interactions with infiltrating immune cells and endothelial cells, thereby promoting sustained inflammation and tissue remodeling 17.

The primary challenge in studying ICI myocarditis lies in its spatial and cellular heterogeneity. Histological examinations often reveal a patchy, non-uniform distribution of inflammatory infiltrates, where lesions are interspersed with healthy tissue 18. Bulk RNA sequencing of cardiac biopsies risks diluting the signal of rare, pathogenic immune clones within the larger pool of healthy cells. Moreover, the critical pathological event, the physical interaction between an aggressive T-cell and a cardiomyocyte within the tissue niche, is lost in tissue homogenization 19. This limitation underscores the indispensable role of single-cell RNA sequencing (scRNA-seq) and spatial transcriptomics, which together can deconvolute the immune microenvironment and visualize the specific neighborhoods where immune tolerance is breached.

One recent study utilizing scRNA-seq analysis demonstrated that monocyte-derived macrophages were significantly expanded in the hearts of Pdcd1-/- mice, a population enriched in the NOD-like receptor signaling pathway 20. To investigate the dual cardioprotective and antitumor mechanisms of the NLRP3 inhibitor MCC950, researchers integrated scRNA-seq with bulk RNA-seq analyses. In cardiac tissue, MCC950 suppressed recruitment of pathogenic macrophages while favoring recruitment and polarization toward reparative macrophages (resembling M2-like phenotypes). This shift consequently attenuated CD8+ T-cell myocardial infiltration, decreased TNF-α secretion, and disrupted the TNF-α-IL-1β positive feedback loop 20.

These findings highlight the excellent intervention value of NLRP3-targeted therapy in ICI-M and underscore the central role of single-cell sequencing in elucidating the therapeutic mechanism. Single-cell sequencing technology has systematically revealed the key regulatory role of specific immune populations in the pathogenesis of ICI-M, laying a theoretical foundation for developing precise therapies that achieve both cardiac protection and sustained antitumor efficacy.

## 3. The Multi-omics Toolbox: Systemic Decryption of Cardiotoxicity

**Figure 1:**
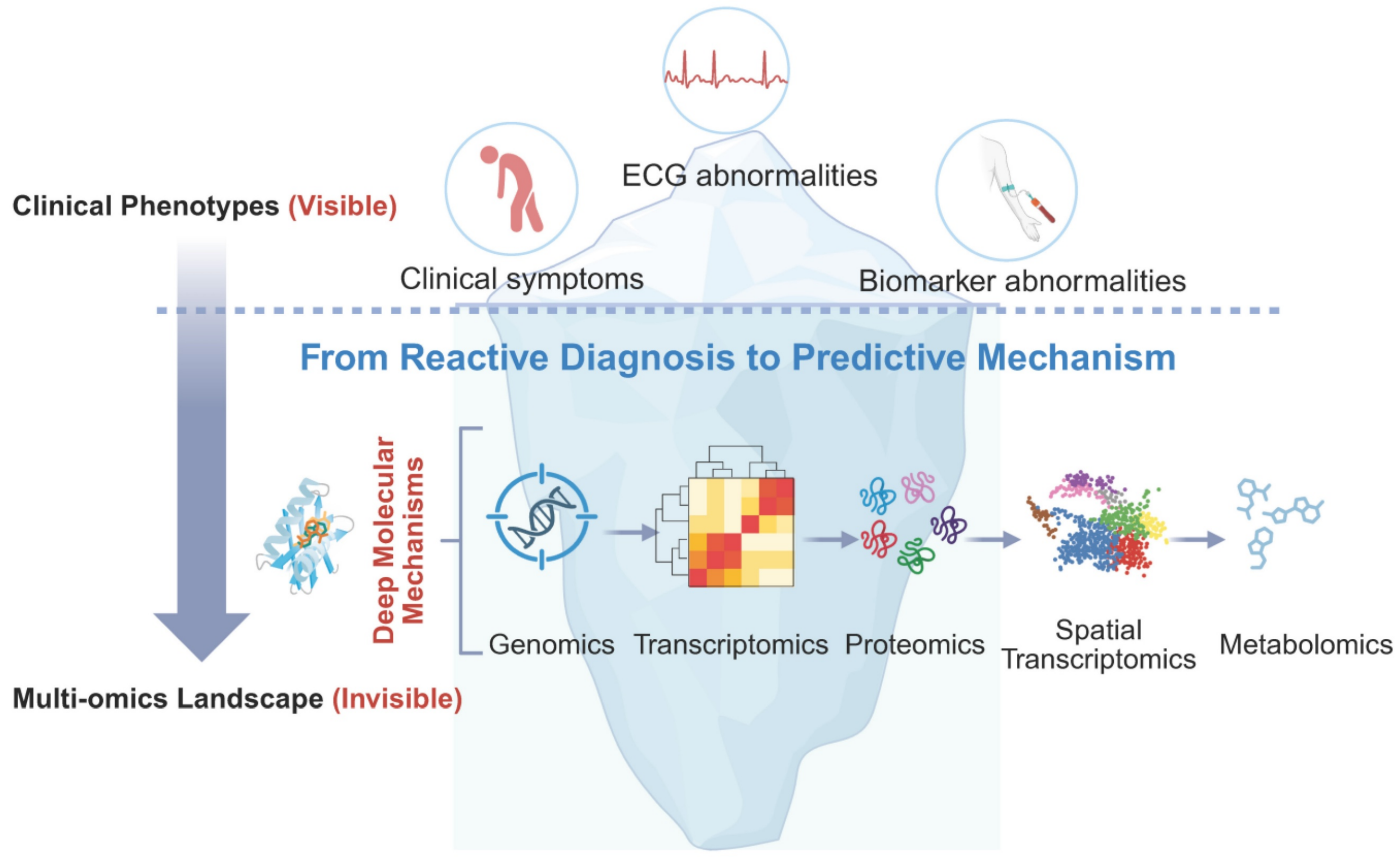
Iceberg metaphor illustrating the shift from traditional clinical indicators to deeper molecular mechanisms in cardio-oncology through multi-omics.

Having established the clinical spectrum, the critical scientific challenge is to deconstruct the molecular heterogeneity that underpins these phenotypes. A specific anthracycline dose may trigger heart failure in one patient while sparing another, and a kinase inhibitor may rewire the cardiac signaling network beyond its intended target. Addressing these questions requires a shift from observing organ-level dysfunction to investigating systemic molecular networks through multi-omics technologies. As illustrated by the iceberg metaphor (Figure 1), conventional clinical indicators capture only the “tip” of cardiotoxicity, whereas deeper molecular programs shape susceptibility, progression, and late outcomes. This framework motivates the subsequent sections, which transition from clinical phenotypes to layered mechanistic pathways and omics-based interrogation.

### 3.1 Genomics: Decoding Genetic Susceptibility and the Second Hit

The observation that cardiotoxicity is not strictly dose-dependent suggests a significant role for germline genetic variation. For decades, the search for genetic predictors was limited to candidate gene studies, which yielded inconsistent results. However, the advent of unbiased Genome-wide association studies (GWAS) has transformed this landscape, identifying robust pharmacogenomic markers that govern individual susceptibility.

One of the most landmark discoveries in this domain involves the RARG gene (Retinoic Acid Receptor Gamma). A seminal GWAS published in Nature Genetics identified that a specific nonsynonymous variant (rs2229774) in RARG dramatically increases susceptibility to anthracycline-induced cardiotoxicity (Odds Ratio = 4.7) 21. Mechanistically, this variant fails to repress Top2b expression, thus leaving the cardiomyocyte genome “open” and hypersensitive to doxorubicin-induced DNA double-strand breaks. Similarly, variants in the solute carrier transporter gene SLC28A3 have been validated as protective, effectively reducing the intracellular accumulation of the drug 22.

These findings support a “Two-Hit Hypothesis”: chemotherapy provides the environmental stress (First Hit), while the patient's germline genetic architecture (Second Hit) determines the threshold for catastrophic failure. Integrating these variants into a polygenic risk score (PRS) represents the first tangible step toward “Precision Cardio-oncology,” enabling clinicians to stratify patients before the first infusion.

### 3.2 Proteomics and Phosphoproteomics: The Functional Execution

While genomics provides the blueprint, proteins serve as the functional effectors. Transcriptional changes do not always correlate with protein abundance due to translational regulation and degradation rates. This is particularly relevant for Type II toxicities (Targeted Therapies), which are primarily driven by post-translational modifications (PTMs) rather than gene expression changes. Accordingly, proteomics captures changes in protein abundance and turnover, whereas phosphoproteomics provides a direct readout of signaling activity through dynamic phosphorylation events that often precede overt functional decline. 23

Kinase inhibitors (e.g., Sunitinib, Sorafenib) are broad-spectrum agents that target a wide range of kinases beyond the tumor. High-throughput phosphoproteomics has shown that these drugs can rapidly rewire the cardiac signaling network. For example, “off-target” inhibition of AMPK (AMP-activated protein kinase) or ribosomal S6 kinase (RSK) impairs the heart's ability to respond to hemodynamic stress 24. Beyond AMPK/RSK, phosphoproteomic studies can reveal coordinated perturbations across major cardiomyocyte stress-response and growth pathways (e.g., PI3K-AKT, MAPK/ERK, mTOR/S6, and Ca²⁺-handling networks), providing a mechanistic explanation for contractile dysfunction, impaired energetic adaptation, and maladaptive remodeling under targeted therapies 25.

By mapping these phosphorylation profiles, researchers can distinguish between non-specific kinase inhibition and critical toxic events, potentially guiding the development of more selective, cardio-safe kinase inhibitors. Importantly, phosphoproteomics can also support the inference of upstream kinase activities from substrate phosphorylation patterns, enabling “pathway-level” toxicity signatures that may generalize across drugs with different nominal targets. When integrated with transcriptomics and metabolomics, these profiles help resolve whether observed changes reflect primary signaling disruption (Type II-like) versus downstream remodeling secondary to injury.

Practical limitations remain. Cardiac phosphoproteomics requires careful control of pre-analytical variables (ischemia time, tissue handling, and batch effects), and depth of coverage is influenced by sample amount and enrichment strategies. Moreover, phosphorylation changes are highly context-dependent (dose, exposure time, and hemodynamic state), making standardized experimental designs essential for cross-study comparability. Nonetheless, expanding high-resolution proteomic and phosphoproteomic profiling—particularly in time-resolved models and well-phenotyped patient cohorts—will be pivotal for identifying actionable signaling nodes and developing mechanism-informed cardioprotective strategies 26.

### 3.3 Metabolomics: Mitochondrial Reprogramming and Energy Starvation

The heart is the most metabolically demanding organ in the body, consuming vast amounts of ATP primarily through fatty acid oxidation (FAO). Metabolomics has proven particularly instrumental in characterizing the “metabolic collapse” that precedes contractile dysfunction.

Anthracyclines induce profound metabolic reprogramming. Recent studies using untargeted metabolomics have shown that doxorubicin does not merely damage mitochondria structurally, but fundamentally alters substrate utilization—suppressing FAO and forcing the heart into a maladaptive reliance on glycolysis. A pivotal study highlighted this mechanism, showing that doxorubicin treatment drastically reduces the levels of key FAO intermediates 27. Crucially, the study provided a translational breakthrough: the use of SGLT2 inhibitors (empagliflozin) was found to rescue this metabolic defect, not by changing glucose handling, but by adaptively shifting cardiac metabolism toward ketone body utilization.

This exemplifies the power of metabolomics: it moved the field from a descriptive observation (“mitochondria appear damaged”) to a functional intervention (“restore metabolic flux”). Furthermore, plasma metabolomics is currently identifying circulating metabolites, such as specific acylcarnitines or lipid species, that may serve as “liquid biopsies” for early mitochondrial distress, potentially outperforming Troponin I in sensitivity 28.

### 3.4 Mechanisms of Late-Onset Cardiotoxicity: Beyond Epigenetic Memory

A significant clinical feature of cardiotoxicity is its latency; heart failure may manifest years, or even decades, after cancer cure. This suggests a mechanism of “biological memory,” where an early toxic insult initiates a progressive decline in cardiac function. Within this framework, epigenomics, encompassing DNA methylation, histone modifications, and non-coding RNAs, provides a pivotal molecular basis for this phenomenon 29. Even after the offending chemotherapy agent is cleared from the body, the epigenetic landscape of the cardiomyocyte may remain permanently “scarred.” For instance, anthracycline exposure can induce widespread and time-dependent changes in cardiomyocyte chromatin accessibility, which are accompanied by transcriptional remodeling. Such epigenetic reprogramming may persist beyond drug clearance and has been proposed to contribute to long-term vulnerability, including pathways related to mitochondrial metabolism and excitation-contraction coupling 30, 31.

However, this molecular memory operates alongside irreversible structural and physiological changes that collectively drive late-stage decompensation. Unlike tissues with high regenerative capacity, the heart suffers from the irreversible loss of post-mitotic cardiomyocytes during acute exposure. This depletion of the functional pool forces remaining myocytes into chronic compensatory hypertrophy, which eventually becomes maladaptive. This process is compounded by interstitial fibrosis, where the expansion of the extracellular matrix disrupts electrical conduction and increases ventricular stiffness 32. Furthermore, persistent mitochondrial dysfunction, driven by impaired mitophagy and accumulated mitochondrial DNA (mtDNA) damage, creates a state of chronic oxidative stress that self-perpetuates long after drug cessation 33, 34.

These local cellular changes are further exacerbated by chronic systemic neurohormonal activation, including the sustained up-regulation of the renin-angiotensin-aldosterone system (RAAS) and the sympathetic nervous system, which drives progressive ventricular remodeling 35, 36. Therefore, late-onset cardiotoxicity is best understood as a cumulative process where epigenetic reprogramming, structural loss, and systemic signaling converge to tip the heart from compensated function into clinical failure. Mapping these multi-layered marks offers not only a future avenue for potentially “erasing” the memory of toxicity but also highlights the need for early interventions that target remodeling and neurohormonal pathways to restore myocardial resilience 37.

## 4. The Frontier: High-Resolution Mapping of the Cardiac Microenvironment

The transition from bulk tissue analysis to high-resolution omics represents the most significant paradigm shift in cardio-oncology. We are moving away from viewing the heart as a homogeneous syncytium toward understanding it as a complex, multicellular ecosystem. This section delineates how single-cell and spatial technologies are resolving the cellular heterogeneity and topological architecture of cardiotoxicity. However, while single-cell sequencing excels at defining cellular diversity, the necessary tissue dissociation shatters the organ's spatial architecture, resulting in the irreversible loss of the physical coordinates and microenvironmental 'niches' that are essential for understanding localized cardiotoxic events.

### 4.1 Single-Cell RNA Sequencing: Unmasking Heterogeneity

Traditionally, bulk RNA sequencing averaged gene expression across the entire ventricle, effectively drowning out the signals from rare but critical cell populations. However, the heart is a mosaic of cardiomyocytes, endothelial cells, fibroblasts, and resident immune cells, each responding distinctively to therapeutic stress. scRNA-seq has allowed us to deconvolute this complexity, revealing that “cardiotoxicity” is often a sum of cell-type-specific failures rather than a uniform global event.

A prime example of this resolution is found in the mechanism of ICI-associated myocarditis. For years, the specific drivers of this fulminant condition remained elusive. Recent scRNA-seq profiling has now definitively identified that the inflammatory infiltrate is not stochastic but driven by the clonal expansion of specific CD8^+^ cytotoxic T cells (expressing high levels of *Gzmb* and *Nkg7*) alongside pro-inflammatory macrophages 38. Crucially, scRNA-seq allows for the analysis of ligand-receptor pairs, suggesting that these immune subsets do not merely exist in parallel but actively coordinate tissue destruction through specific checkpoints (e.g., CCR5/CCL5 axes). Furthermore, in the context of anthracycline toxicity, single-cell data have unmasked the role of non-myocytes, showing that endothelial cells 39 and fibroblasts 40 undergo distinct transcriptional trajectories, such as early senescence and pro-fibrotic activation, long before cardiomyocyte apoptosis becomes detectable. This confirms that the “first hit” of toxicity often occurs in the supporting stroma, a nuance completely invisible to bulk analysis.

Beyond direct cardiomyocyte injury, many systemic anticancer therapies exert significant vascular toxicity, which often precedes or exacerbates myocardial dysfunction. Multi-kinase inhibitors targeting the vascular endothelial growth factor (VEGF) pathway, such as Sunitinib and Sorafenib, are well-documented to induce endothelial dysfunction, arterial hypertension, and vasospasm 41, 42.

From an omics perspective, these agents trigger a pro-inflammatory and pro-thrombotic shift in the vascular microenvironment. Spatial transcriptomics and single-cell landscapes have begun to resolve how VEGF inhibition disrupts the NO-mediated localized vasodilation and promotes capillary rarefaction within the myocardium. This “vascular hit” increases afterload and reduces coronary microcirculatory reserve, creating a synergistic state of failure with direct myocyte toxicities 43. Recognizing the endothelial compartment not merely as a structural scaffold but as a primary toxicological target is essential for a holistic understanding of the cardio-vascular side effects of targeted therapies 42.

### 4.2 Spatial Transcriptomics: The Importance of Localization

While scRNA-seq provides a comprehensive profile of the gene expression in the cardiotoxic heart, it requires tissue dissociation, which results in the loss of spatial context. This limitation is critical, as pathology is rarely homogeneous and is instead topologically organized. Spatial Transcriptomics (ST) addresses this challenge by mapping gene expression directly onto the tissue architecture, enabling the creation of a “Spatial Atlas of Cardiotoxicity.”

**Figure 2:**
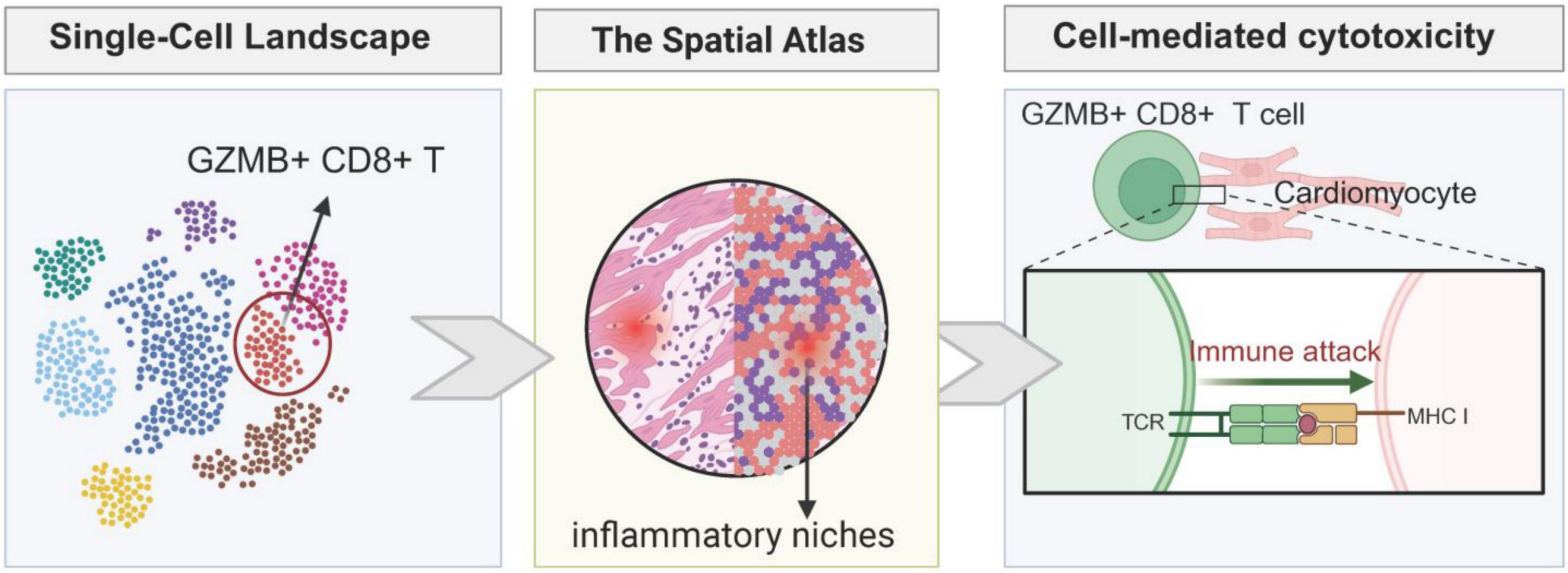
Single-cell and spatial atlas revealing the microenvironment of ICI myocarditis and cell-mediated cardiotoxicity.

The strength of ST lies in its ability to resolve the interactions within tissue niches. In ICI myocarditis, pathology typically manifests as patchy infiltrates. ST allows for precise localization of these lesions, enabling the identification of whether the damage is perivascular, indicating blood-borne immune cell infiltration, or more diffuse within the endocardium. By analyzing the spatial relationship between immune cells and cardiomyocytes, ST can generate interaction scores that reflect the proximity and interaction between these cells. Recent studies using spatial platforms have visualized the immunological synapse *in situ*, demonstrating that pathogenic T-cells specifically cluster around cardiomyocytes with high levels of MHC-I expression, providing direct evidence of the immune response targeting specific cardiac cells within the tissue microenvironment 44. As depicted in Figure 2, this spatial delineation of the injury site is essential for distinguishing genuine myocarditis from bystander inflammation.

### 4.3 Spatial Metabolomics (MALDI-IMS): Visualizing Chemistry

Beyond gene expression, the actual distribution of the drug and its metabolic consequences defines the landscape of toxicity. Matrix-Assisted Laser Desorption/Ionization Mass Spectrometry Imaging (MALDI-IMS) has emerged as a powerful tool for Spatial Metabolomics, allowing for the label-free visualization of molecules directly on histological sections 45.

This technology provides the most intuitive evidence for Pharmacokinetics/Pharmacodynamics (PK/PD) at the tissue level. Historically, we assumed that systemic drug administration resulted in homogenous cardiac exposure. However, MALDI-IMS has revealed that drugs like doxorubicin accumulate heterogeneously, forming distinct “hotspots” within the myocardium. Crucially, these drug-rich regions spatially correlate with specific metabolic signatures, such as zones of ATP depletion or lipid peroxidation 46. By overlaying the drug distribution map with the metabolic damage map, we can directly link local drug retention to local mitochondrial failure. This “visual chemistry” moves the field beyond serum biomarkers, offering a direct window into the chemical warfare occurring within the myocardial tissue.

### 4.4 Current Constraints and Methodological Challenges

Despite the transformative potential of multi-omics and spatial technologies, several critical constraints hinder their routine implementation in cardio-oncology (Table 1). First, a significant hurdle is the lack of standardized analytical pipelines across different spatial platforms (e.g., 10x Visium, Xenium, and Slide-seq). Variations in data normalization, cell-type deconvolution, and spatial clustering algorithms often lead to platform-specific biases, complicating cross-study comparisons 47. Second, while *in situ* hybridization-based methods offer high resolution, many capture-based spatial transcriptomics methods still suffer from limited cellular resolution (e.g., Visium's 55-μm spots), which may capture mixed signals from multiple cell types (cardiomyocytes, fibroblasts, and immune cells) in the dense cardiac tissue. Third, the prohibitive cost of high-throughput spatial sequencing remains a barrier to large-scale longitudinal studies. Finally, there is a notable absence of prospectively validated omics biomarkers in clinical cardio-oncology. Most current findings remain at the discovery stage, and the translation of complex molecular signatures into robust, cost-effective clinical diagnostics requires rigorous validation in multi-center prospective cohorts. Addressing these bottlenecks is essential to transition from “bench-side discovery” to “bedside precision medicine”.

## 5. Integration: Multi-omics Fusion and Artificial Intelligence

The generation of high-dimensional datasets, ranging from genomic susceptibility to spatial metabolic maps, brings us to a critical crossroads. Relying on any single omics layer is akin to the parable of the blind men and the elephant; each technology provides a partial view of the truth, but none alone can offer a complete understanding of cardiotoxicity. To transition from fragmented insights to a comprehensive understanding, we must embrace Trans-omics, a methodology that integrates individual layers to reconstruct the global molecular networks that govern the transition from health to disease.

### 5.1 From Silos to Systems: Trans-omics and Network Pharmacology

The biological response to cancer therapy is not linear, but rather reticular. A drug does not merely target a specific molecule; it perturbs an entire network. Trans-omics analysis integrates biological information flow, from the genome (potential) to the transcriptome (execution), and ultimately to the metabolome and proteome (phenotype), to uncover causal relationships that are invisible when studied in isolation. This approach is effectively operationalized through Network Pharmacology.

By constructing “Drug-Target-Pathway-Phenotype” networks, researchers can map the topology of toxicity. For instance, while a Tyrosine Kinase Inhibitor (TKI) may be designed to target a tumor-specific kinase, network analysis can reveal its off-target binding to cardiac kinases with structural homology. This framework enables the visualization of the “Cardiotoxicity Interactome,” predicting adverse events based on the proximity of drug targets to essential cardiac survival modules 48. Rather than viewing side effects as random occurrences, network pharmacology redefines them as predictable consequences of network perturbation, providing a rational foundation for designing “network-neutral” drugs that minimize harm to the heart.

### 5.2 The Computational Engine: AI and Multimodal Fusion

The vast volume and heterogeneity of these data—ranging from unstructured pixel data in spatial imaging to quantitative measures in metabolomics—exceed human cognitive processing capabilities. In this context, Artificial Intelligence (AI) and Machine Learning (ML) become indispensable.

**Figure 3:**
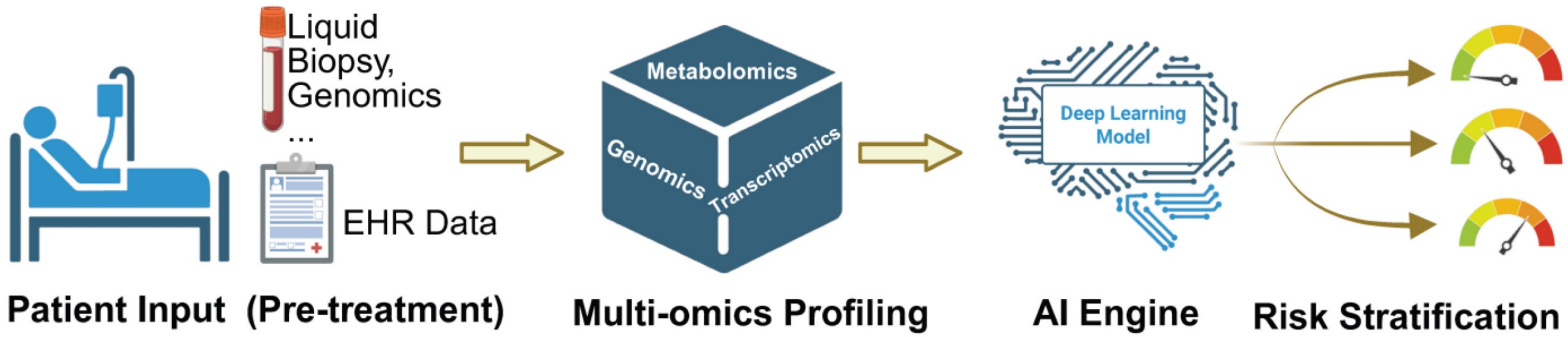
AI-integrated clinical workflow for precision cardio-oncology, guiding personalized treatment and risk stratification.

The frontier lies in Multimodal Data Fusion. Advanced deep learning algorithms can now process disparate data streams simultaneously to develop precision risk prediction models. For example, an AI model can integrate a patient's polygenic risk score (from genomics), their baseline metabolomic profile (from metabolomics), and subtle textural features from an echocardiogram (from radiomics) to predict the likelihood of heart failure with accuracy far surpassing that of traditional regression models 49. The concept of “Digital Twins” represents a forward-looking framework in which multimodal patient data could be integrated to simulate individualized therapeutic responses. While promising, this approach remains largely conceptual in cardio-oncology and will require prospective validation before clinical implementation. To clarify the translational pathway, Figure 3 outlines an AI-integrated clinical workflow that progresses from pre-treatment patient inputs to individualized risk stratification. Baseline clinical characteristics and EHR-derived features, complemented when available by liquid biopsy and genomic testing, are integrated with multi-omics profiling (e.g., genomics, transcriptomics, and metabolomics) to capture latent molecular vulnerability beyond conventional markers. These multimodal features are subsequently synthesized by an AI engine into patient-specific risk estimates, enabling clinically actionable stratification that may inform surveillance intensity, early cardioprotective strategies, and therapeutic decision-making.

However, the deployment of AI-driven models in cardio-oncology raises important considerations regarding interpretability and accountability. Many deep-learning architectures operate as “black boxes,” potentially limiting clinician trust and regulatory approval. Future development must therefore prioritize explainable AI frameworks, transparent model validation, and clearly defined lines of clinical responsibility to ensure safe and ethically sound implementation.

## 6. Clinical Implications: From Bench to Bedside

The ultimate validation of multi-omics does not lie in the mere accumulation of datasets, but in their translation into tangible clinical benefits. As we decode the molecular lexicon of cardiotoxicity, we are witnessing a paradigm shift from reactive management—treating heart failure after it occurs—to proactive precision care. However, most omics-driven strategies in cardio-oncology remain at the discovery-to-early validation stage, and only a subset has entered investigational clinical use. This translation is manifesting primarily in two key areas: the next generation of biomarkers and mechanism-guided cardioprotection.

### 6.1 Liquid Biopsy: Emerging Biomarkers for Early Detection

While Troponin I and BNP remain clinical gold standards, they are inherently limited by their release kinetics, typically reflecting established cellular injury. The concept of “Liquid Biopsy” offers a revolutionary alternative. By analyzing the circulating “ome,” we can potentially detect subclinical distress signals long before structural damage occurs. At present, these approaches are not routine standards of care and require prospective validation, assay harmonization, and demonstration of incremental clinical utility beyond established biomarkers.

Circulating cell-free DNA (cfDNA) represents the forefront of this innovation. Unlike proteins, cfDNA carries methylation signatures that are tissue-specific. Recent studies using methylation-sensitive sequencing have demonstrated the ability to distinguish cfDNA specifically originating from dying cardiomyocytes, as opposed to endothelial cells or fibroblasts 50. This specificity enables the research-level quantification of cardiac tissue loss with sensitivity that surpasses traditional enzymatic biomarkers. Furthermore, exosomes, extracellular vesicles transporting microRNAs and proteins, serve as “messengers” from the cardiac microenvironment. Profiling the exosomal cargo, such as specific miRNA panels like miR-1 or miR-133a, provides a real-time window into the heart's transcriptional state, offering a non-invasive method to monitor “molecular stress” in the myocardium during chemotherapy cycles 6. Nevertheless, variability in pre-analytical handling, platform-specific measurement, and cohort heterogeneity currently limit direct clinical adoption.

Importantly, circulating systemic biomarkers and *in situ* tissue pathology should not be viewed as competing layers, but as complementary scales of the same biological process. Circulating cfDNA, metabolites, or exosomal signatures may function as accessible surrogates of spatially organized tissue injury, whereas single-cell and spatial profiling provide the mechanistic ground truth needed to interpret and validate these systemic signals. In this framework, *in situ* atlases inform biomarker selection, while systemic markers enable longitudinal monitoring without repeated tissue sampling.

### 6.2 Mechanism-Guided Cardioprotection

Beyond diagnosis, multi-omics provides the rationale for targeted interventions. The traditional “one-size-fits-all” approach to cardioprotection (e.g., universal ACE inhibitors) is being replaced by strategies tailored to the specific molecular perturbations induced by cancer therapies. Yet, for most proposed interventions, mechanistic plausibility currently outpaces prospective clinical evidence, and translation should be viewed as evolving rather than established.

For example, metabolomic studies have shown that doxorubicin suppresses fatty acid oxidation, leading to an energy-starved heart. This finding has directly supported the repurposing of SGLT2 inhibitors. By promoting ketone body utilization, these agents bypass the metabolic blockade, offering a mechanism of rescue that differs from hemodynamic unloading. Similarly, in the context of ICI myocarditis, the discovery of cytokine-mediated toxicity through scRNA-seq (e.g., via the JAK-STAT pathway) has facilitated the off-label use of targeted immunomodulators like Abatacept (CTLA-4 agonist) or Ruxolitinib (JAK inhibitor) for refractory cases. This shift in clinical practice moves away from empiric high-dose steroids toward precision immunomodulation.

## 7. Conclusion and Future Perspectives

We stand at a pivotal moment in the history of cardio-oncology. The era of defining cardiotoxicity solely through the lens of echocardiographic ejection fraction is drawing to a close. As this review has delineated, cancer therapy-induced heart disease is a multi-dimensional pathology driven by complex interactions among genetic susceptibility, metabolic reprogramming, immune dysregulation, and spatial microenvironmental disruption. Importantly, these mechanisms are not uniform across therapies: anthracyclines, immune checkpoint inhibitors, and targeted kinase inhibitors each perturb distinct molecular circuits, yet converge on shared endpoints of remodeling, energetic failure, and loss of functional reserve.

The integration of multi-omics and spatial technologies is not merely refining our current knowledge; it is redefining the taxonomy of the disease itself. Single-cell and spatial atlases are beginning to reveal that “cardiotoxicity” is often the cumulative result of cell-type-specific failures—spanning cardiomyocytes, endothelial cells, fibroblasts, and immune populations—and that many lesions are topologically organized within niches that are invisible to bulk profiling. In parallel, proteomics and phosphoproteomics provide a functional layer that captures signaling rewiring and post-translational control, particularly relevant for Type II toxicities driven by targeted therapies. Together, these approaches enable a mechanistic, systems-level view in which patient-to-patient variability becomes an interpretable biological phenomenon rather than unexplained clinical noise.

We are moving toward a future of “Precision Cardio-Oncology,” where a patient's journey begins not with the first infusion, but with pre-treatment risk stratification that integrates clinical features with omic vulnerability signatures. In this envisioned future, a pre-treatment cardiac risk panel—combining polygenic risk scores, baseline metabolomics, and selected circulating biomarkers—could inform regimen selection, surveillance intensity, and early cardioprotective strategies, helping ensure that the cure for cancer does not come at the cost of the heart. AI-enabled integration of multimodal data further provides a pragmatic route to translate high-dimensional signals into clinically actionable categories, with the long-term aspiration of patient-specific modeling frameworks.

At the same time, several bottlenecks must be addressed to achieve clinical impact. Cross-platform standardization, assay reproducibility, cost, and computational scalability remain substantial barriers, and most proposed omics biomarkers still lack rigorous prospective validation in multi-center cohorts. Ethical and practical considerations—data governance, interpretability, and equitable access—will also shape how rapidly these tools can be deployed. Thus, the near-term priority is not only discovery but disciplined translation: harmonized pipelines, well-phenotyped longitudinal cohorts, and prospective studies that test whether omics-guided interventions measurably improve outcomes.

Despite its promise, the implementation of multi-omics in routine practice faces substantial barriers, including cross-platform standardization, assay reproducibility, regulatory approval pathways, reimbursement considerations, and data governance. Furthermore, many proposed biomarkers and risk models require validation in prospective, multi-center cohorts before regulatory endorsement and widespread clinical integration.

The trajectory, however, is clear. The microscope is increasingly complemented—and in some contexts replaced, by the sequencer, the mass spectrometer, and spatial imaging platforms. By embracing multi-omics, spatial context, and integrative analytics, cardio-oncology is poised to move from reactive detection of dysfunction to proactive prevention grounded in mechanism. In doing so, we may finally open the black box of cardiotoxicity and deliver truly individualized cardiovascular care for cancer survivors.

## Figures and Tables

**Table 1:** Comparative overview of major spatial omics platforms

Platform	Technology type	Resolution	Capacity	Key constraints
10x Visium	Capture-based ST (sequencing)	55 µm spot	Whole transcriptome	Spot mixing; pipeline variability; cost/scalability
Stereo-seq	Chip-based capture (sequencing)	0.5-1 µm bin*	Whole transcriptome	Compute-heavy; bin-depth trade-off; limited standardization
Slide-seq (v2)	Bead-based capture (sequencing)	~10 µm	Whole transcriptome	Lower sensitivity; deep sequencing; batch effects
CosMx	*In situ* hybridization (imaging)	Subcellular	Targeted (≤ ~6000 genes)	Targeted only; long imaging; high cost
Xenium (10x)	*In situ* hybridization (imaging)	Subcellular	Targeted (hundreds-thousands)	Targeted only; panel required; high cost
DBiT-seq	Microfluidic barcoding	~10-50 µm (design-dependent)	Transcriptome ± proteins (assay-dependent)	Complex setup; limited standard pipelines; throughput constraints
MALDI-IMS	Mass spectrometry imaging	10-50 µm	Metabolites & lipids	No transcriptome; ID/annotation challenging; specialized instrumentation

* Nominal resolution depends on bin size and sequencing depth.
